# "Life mapping" exploring the lived experience of COVID-19 on access to HIV treatment and care in Malawi

**DOI:** 10.12688/gatesopenres.15927.2

**Published:** 2024-09-24

**Authors:** Jane Harries, Ruby T. Zolowere, Khokhelwa Zokwana, Krista Lauer, Jelena Bozinovski, Solange L. Baptiste

**Affiliations:** 1 University of Cape Town, Rondebosch, Western Cape, 7700, South Africa; 2 International Treatment Preparedness Coalition, Johannesburg, 2196, South Africa

**Keywords:** Qualitative Research, Participatory Research, COVID-19, HIV treatment, Malawi

## Abstract

**Background:**

The COVID-19 pandemic in Malawi exacerbated, existing public health challenges including access to HIV treatment and care services. “Life Mapping,” a component of the Citizen Science community-led project in Malawi, documented the lived experiences and perspectives of people living with HIV in the context of COVID-19.

**Methods:**

Citizen Science Life Maps is a three-year qualitative, longitudinal project utilizing collaborative and participatory research methods through digital storytelling to document peoples’ daily lives. Twenty participants living with HIV were recruited between 2022 and 2023 in two central regional districts of Malawi and two urban areas. The participants were given mobile smart phones to document the impact of COVID-19 on HIV prevention and treatment services, HIV treatment literacy, mental health and the COVID -19 vaccine. Data was analyzed using a thematic analysis approach.

**Results:**

Access to HIV prevention and treatment slowly recovered yet introducing multi-month anti- retroviral dispensing raised concerns. In the absence of mental health care services, participants were resourceful in seeking alternative ways to deal with mental health. However, state sponsored violence in relation to COVID-19 public health measures impacted negatively not only on mental well-being but also on HIV treatment adherence. Whilst most recognized the importance of the COVID-19 vaccine, especially for people living with HIV, myths, misinformation, and conspiracy theories around the vaccine persisted especially religious themed misinformation.

**Conclusions:**

The relationship between misinformation and COVID-19 vaccine hesitancy is complex and medical and scientific approaches may not be sufficient to prevent misinformation. Fear and misinformation are likely attributed to global uncertainty during the pandemic and the speed at which vaccines were developed with minimal opportunity to prepare global communities.

## Background

 In March 2020, the World Health Organization (WHO) declared COVID-19, the disease caused by the novel strain of coronavirus, severe acute respiratory syndrome coronavirus 2 (SARS-CoV-2), a global pandemic ^1^. The rapid spread of SARS-CoV-2 led most countries to declare a state of emergency and implement strict measures to contain the virus. In February 2024, a total of 774 million COVID-19 cases had been reported worldwide including 7 million reported deaths ^2^. In May 2023, the WHO declared an end to COVID-19 as a public health emergency ^3^. While COVID-19 is no longer considered a global health emergency, its unprecedented impact on healthcare, economies and people’s lives persists.

The response to the COVID-19 pandemic resulted in severe healthcare service disruptions globally, affecting especially low- and middle-income countries with fragile health systems and limited resources ^1, 4, 5^.

Malawi declared a state of national disaster due to the COVID-19 pandemic in March 2020 and registered its first confirmed case in April 2020 ^6^. Restrictive measures were put in place to prevent the spread of the virus and mitigate COVID-19’s impact, which included travel and movement restrictions, mandatory use of face masks in public places and bans on social gatherings ^7^. By December 2023, Malawi had reported 89,161 cases and 2,686 confirmed deaths ^8^.

The COVID-19 pandemic in Malawi posed new challenges and exacerbated existing public health challenges. Similar to other countries, COVID-19 restrictive measures in Malawi negatively impacted access to HIV prevention and treatment services ^5, 7^. Malawi has one of the highest burden of HIV/AIDs in Africa with an estimated HIV prevalence among adults (15 – 49 years) of 7.1% and an estimated 1 million people living with HIV (PLHIV) ^9^. Despite the high burden of HIV infections, Malawi has made notable progress towards the UNAIDS 95-95-95 ^10^ targets for HIV control (91% of PLHIV knowing their status, 86% of whom are on ART, and with 81% of those on ART achieving virologic suppression) ^7^. However, these achievements could be compromised with the disruption of essential health services including HIV treatment services ^11^. During the implementation of COVID-19 restrictions, there was a decrease of 31.9% in HIV testing and a corresponding 22.8% decline in the diagnosis of PLHIV in Malawi. However, there have been recent reports of recovery in delivery of HIV services ^5, 7^. The uneven recovery patterns observed across populations receiving HIV services seems to suggest that children, young people and men are being left behind ^7, 12^.

Beyond access to HIV services, the COVID-19 pandemic further exacerbated the vulnerability of PLHIV emerging from structural and socio-economic conditions such as high poverty levels, stigma and poor mental health ^13– 15^. The lived experiences of PLHIV during the COVID-19 pandemic and the social impact of restrictive measures, social isolation and changes in health care delivery on peoples everyday lives and well-being has been overlooked in the African context, including Malawi, and much remains to be explored ^13^.

Community-based participatory research has proven to be a valuable approach to explore public health conditions and the social determinants of health, while also enhancing the participation of marginalized and vulnerable populations ^16– 18^. In this method, community members are placed as partners in all phases of the research process, guided by frameworks that promote participation, individual voice and representation ^16, 19^. The implementation of this method aims primarily to promote social change, improve health outcomes and educate ^18^.

Against this backdrop, the International Treatment Preparedness Coalition (ITPC) launched COVID-19 Citizen Science in 2022, a community-led project documenting perspectives, experiences and advocacy priorities among people living with HIV in Malawi and South Africa with the overall goal of improving health outcomes. Citizen Science moves from models of “data extraction” to “data democracy” by combining community-led monitoring (CLM), operational research, and an innovative research methodology that we have called “Life Mapping”, which uses collaborative and participatory visual and textual media tools to document people’s everyday life experiences of HIV prevention, treatment, and care ^20^.

Community led monitoring in this study context, can be described as a community monitoring and research initiative that gathers data on access to and quality of HIV treatment. CLM aims to streamline and standardize HIV treatment access data collected by communities and uses this data to guide advocacy efforts and promote accountability ^20^. While the findings and impact of the CLM effort have been explored elsewhere ^21, 22^, this article explicitly explores the latter component of the project, termed “Life Maps.”

## Methods

### Study design

The Citizen Science “Life Maps” project is a three-year qualitative, longitudinal project currently being implemented in South Africa and Malawi. In this article we report on the findings in Malawi only. We used a collaborative and participatory research method utilizing digital storytelling to document peoples’ lived experiences during the COVID-19 pandemic with a focus on access to HIV treatment and prevention. Each participant used a mobile smart phone to capture photos and record narratives. This method provided participants with the opportunity to not only avoid frequent face to face contact with study team members (which was important during the height of COVID-19 restrictions) but also allowed for depth and reflection beyond more traditional qualitative interviews. We used and adapted the term “Life Mapping” informed by a life history approach in which participants were able to narrate their everyday life experiences through textual and visual presentations on diverse topics during and in the aftermath of the COVID-19 pandemic ^23^.

### Research setting and study population

Data was collected between May 2022 to June 2023 in two central regional districts of Malawi, Kasungu and Dedza, and two urban areas, Lilongwe and Blantyre. Study participants were recruited with the assistance of the Malawi Network of Religious Leaders Living with or Personally Affected by HIV and AIDS (MANERELA +) who are the implementing partners of the broader Citizen Science community led monitoring project in Malawi. Participants were recruited from districts where Citizen Science was being implemented. We employed convenience sampling followed by snowball sampling. Eligibility criteria included people living with HIV, older than 18 years and willing to participate in a three-year study. MANERELA + staff initially identified potential study participants from advocacy groups associated with MANERELA +. This was followed with snowball sampling where enrolled participants further identified potential study participants. In total, 20 participants (10 women and 10 men) were enrolled into the study. The project manager who was conversant in Chichewa (local language) and trained in public health met with eligible study participants and further explained the study and sought informed consent. All participants received a mobile smart phone (Redmi Note 11 Pro +) and participated in a training session about the study including how to use the different digital tools which included short text messaging, voice notes and taking photographs and videos. All participants were provided with monthly data and airtime.

### Data collection

Participatory qualitative research methods were used. The topics for exploration were initially decided a priori, and explored aspects of HIV treatment, encompassing broader issues related to the impact of the COVID-19 pandemic on people’s lives. Topics were initially sent every 2 weeks via text using the Telegram platform. Key topics and themes explored and reported in this paper included: i) Impact of COVID-19 on HIV treatment and prevention services; ii) HIV treatment literacy; iii) mental health including stigma; and iv) the COVID -19 vaccine. Each topic was introduced to participants and then followed by short, open-ended questions to guide the responses. Study participants used their mobile phones to respond to the topic using photographs, videos, voice notes, and text messages. Photographs were taken to document various experiences related to specific topics, such as images depicting phenomena observed at health care facilities or images illustrating strategies for addressing mental health issues.

As this was a longitudinal project, periods of data collection were interspersed with reflective feedback meetings with study participants (in person or remote). Following up from these feedback meetings, the team decided to introduce the topics every 4 weeks which allowed for more reflection and time to provide responses to the topics. At the end of a year of data collection, a meeting was held with participants to obtain input on future topics for exploration. We then adapted and tailored the topics in 2023 to the changing landscape of the COVID-19 pandemic including new topics suggested by participants which included amongst others the COVID-19 vaccine.

### Data analysis

Data analysis included both textual and visual interpretation of data by the study team. All textual data, including captions accompanying the photographs, were transcribed and translated from Chichewa into English by the project manager who is fluent in both languages. All data was coded (manually) and categorized into key issues identified in the topic guides and from the feedback meetings by the authors (JH, RTZ, KZ). Data were further analyzed using a thematic analysis approach, in which main themes and categories were identified and analyzed within and across all forms of data ^24^. The research team met regularly to compare and discuss findings until consensus was reached.

### Ethical considerations

Ethical approval was obtained from the National Health Sciences Research Committee of Malawi (approval number 2267). All study participants were provided with written explanations of the work, and thereafter gave their informed consent via signature. Confidentiality and anonymity were ensured, and all participants were given agreed upon pseudonyms to protect their identity. All data were closely controlled and stored in locked files and password protected computer files. All participants were provided a monthly stipend of 24,500 Malawian Kwacha to compensate for their time. Additional written informed consent for the publication of the participants’ images created for the project was obtained from all participants.

## Results

### Participant characteristics

The mean age for study participants (n=20) was 41.5 years (range 28–65) with equal gender distribution and all were PLHIV. In terms of employment, 5/20 were unemployed with others involved in the informal farming sector and part time work.

In this paper, we report on four broad themes i) impact of COVID-19 on access to HIV treatment and prevention services; ii) health literacy in relation to HIV prevention and treatment; iii) mental health; and iv) the COVID -19 vaccine. These topics were linked to the impact of the unfolding pandemic on peoples’ everyday lives, with a focus on HIV services underscored by broader structural and socio- economic conditions.

### COVID-19 and access to HIV services

We explored the impact of the COVID-19 pandemic on HIV treatment and prevention services including access to HIV self-testing, antiretroviral therapy (ART), and pre-exposure prophylaxis (PrEP) due to the disruption of treatment services during the pandemic.

***Access to HIV self-testing kits.*** A participant recounted how initially during the pandemic HIV testing services were reduced as health care providers focused on public health measures to curb the spread of the virus. With the easing of restrictions, services slowly recovered. He explained:

*“In the initial stages, COVID did impact access to HIV testing services due to the new alignment of "COVID-19 Prevention Protocols" … until people got used to the new guidelines… HIV self-testing kits are now readily available in many health centers”.* Male, 60–65 years, Telegram Text.

In some settings, access to HIV services including the distribution of self-testing kits, were further hindered by fears of mandatory COVID-19 testing and vaccination.

*“People are also afraid to go for tests especially HIV because a lot of people they think that the time they are getting tested for HIV is the same time the hospital takes advantage of testing people for COVID-19. Lastly COVID-19 have affected HIV testing. It was last week I went at Kanyama I was distributing HIV self-testing kits, but some people were refusing to take the kit. Because they were thinking that it was the vaccine for COVID-19”.* Male, 20–30 years, Telegram Text.

***Anti-retroviral therapy (ART) access.*** Although three-and six-monthly ART refills have been a WHO-recommended policy for stable patients since 2016, COVID-19 rapidly sped up implementation of multi-month dispensing (MMD) in sub-Saharan Africa ^25^. While most participants reported that they were receiving their ARVs on a three to six monthly basis, access to the antibiotic sulfamethoxazole and trimethoprim (Bactrim) given to PLHIV with a low immunity was not available.

*“ARV dispensing has changed, in the past for a person who was adhering and had a viral load that was very low, they would give them medication for 6 months now they give for 3 months and in terms of stock outs, Bactrim is only given to those that have a low immunity”*. Female, 30–40 years, Telegram text.

However, for some, the downside of providing ARV’s every 3 to 6 months meant less interaction at health care facilities where issues such as potential side effects could be addressed. A participant was somewhat skeptical of the new dispensing practice especially no longer receiving Bactrim.

*“The ARV supply has also changed before they used to give 1 to 3 months this would help to know fast if there is a problem for example side effects of ARV and they deal with the problem fast but for now due to COVID-19 they give 3 to 6 months for one’s next visit. But for the Bactrim they used to give to patients they have stopped giving”.* Female, 30–40 years, Telegram Text.

Similarly, access to oral PrEP were also interrupted during COVID-19. Malawi National Guidelines for oral PrEP ^26^ provides guidelines as to key service delivery points in public health facilities, however PrEP was mainly available in district and mission hospitals and not in primary health care facilities.

In addition, travel and other restrictions further hampered access to limited PrEP services.

***Socio-economic impact on treatment adherence.*** Broader issues such as access to clean water and increased food prices emerged in relation to the ability to adhere to treatment regimens **.**

Access to clean, drinking water was impacted during the COVID-19 pandemic. Many participants accessed their water through boreholes, however lockdowns impacted on the regular maintenance of boreholes.

A participant explained how prior to COVID-19, families and communities were responsible for cleaning and maintaining their boreholes, however during the height of the pandemic this was interrupted resulting in potentially unsafe drinking water.

*“Access to clean water has affected my family and my community regarding COVID-19 because before COVID-19 we used to take care of our borehole and we had a timetable of cleaning the borehole as one way of hygiene in our community. But all this stopped, and we are no longer cleaning it because people are saying there is COVID-19”*. Female 30–40 years, Telegram Text.

**Figure 1.**
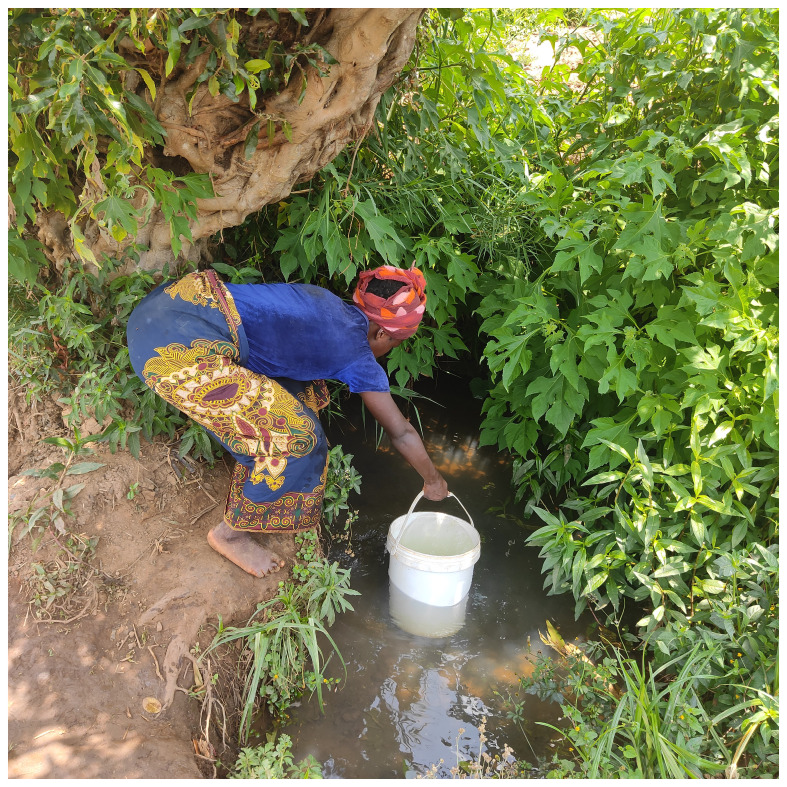
Participant collecting water from a borehole.

The image below Figure 1 depicts challenges of accessing clean drinking water and was captioned “this is where we draw our water from”.

Access to clean water further impacted on the ability to take chronic medication in a timely manner as people spent time looking for alternative sources of water often at distances from their homes.

*“When access to water was a problem, it was indeed a problem for me to adhere to my medication”.* Female 30– 40 years, Telegram Text.

Furthermore, rising food prices led to skipping meals and made it difficult for people to take their medications with food. Poignant images of bare refrigerators captured the challenges faced in taking medications. A participant recounted how food prices had doubled:

 “ *In the past l used to buy 2 trays [of eggs] and a tray was at K 2,600 and one egg was K 100. Now, a tray is K4,600 and an egg is K200 and now l cannot afford to buy eggs, beef and milk”.* Female 30–40 years, Telegram Text.

**Figure 2.**
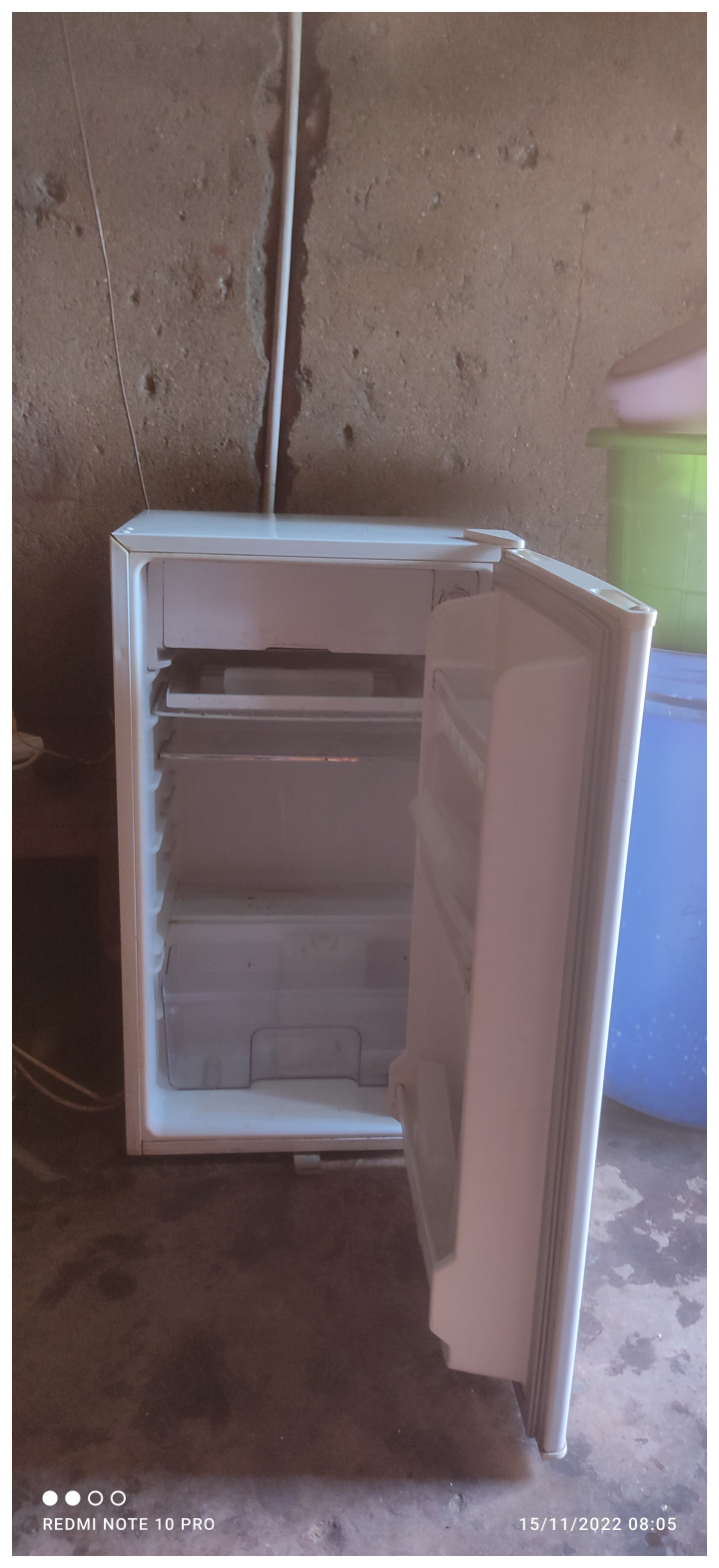
An empty refrigerator.

The below image of “my empty fridge” ( Figure 2) captures the dire situation in relation to access to food.

### Treatment literacy

As part of exploring participants’ understandings of various aspects of their HIV treatment including ART adherence, the concept of undetectable equals untransmittable (U=U) was explored. The U=U global HIV campaign aimed at enhancing ART adherence was being promoted in Malawi at the time. We further explored whether participants knew what an undetectable viral load meant in terms of HIV transmission.

While some participants were not aware of the term U=U they did understand what undetectable viral load and untransmittable meant.

 “ *According to my understanding the meaning of U=U is the first U represents Undetectable the second U represents Untransmittable meaning that if your viral load is in a state of undetectable you can't transmit HIV to anyone else”.* Male, 20–30 years, Telegram Text.

The process of posing the questions around U= U enabled many participants to engage with the concepts of undetectable viral load in relation to HIV transmission and elicited conversations around the importance of ARV adherence in the context of HIV transmission. Posing questions around undetectable viral loads suggests that this was a valuable information sharing exercise as many participants undertook their own research mainly through Google searches.

Apart from U=U we also explored understandings around HIV drug resistance. There were varying responses to HIV drug resistance or what it meant. Some participants understood what it meant whereas others did not fully understand. This would be important to explore further to support and encourage ART uptake and adherence.

*“This means that a person is not taking drugs as prescribed by the doctor. As a result of this the viral load may be higher showing that you’re not adhering to drugs. In other words, we can say that the drugs you are taking are not responding to the disease”*. Female, 40–50 years, Telegram text

Another participant explained that he “ *did not know what it means and the hospital that l receive my medication does not know about this…. Maybe it can mean if you have a viral load that is low, you cannot transmit”.* Male, 20–30 years Telegram text. 

### Mental health

We explored the impact of the COVID-19 pandemic on overall mental health and well-being. Although there were discussions about anxiety, depression and concerns about livelihoods, the overwhelming response was around aggression associated with state sponsored violence regarding enforcing COVID-19 regulations. Frequent narratives about police brutality highlighted not only the impact on people’s mental well-being but also on HIV treatment adherence.

A participant recounted her experience of police aggression for not wearing a face mask and having to pay a bribe to avoid imprisonment.

*“I have been mentally affected by aggression caused by COVID-19, since the coming in of COVID in 2020 and 2021, it caused a lot of changes in people’s lives. … the police would arrest us at the marketplace because we are not wearing a mask. If we are found at a bar, they would arrest us, and it required money to be released”.* Female, 30–40 years, Telegram Text.

She further highlighted the knock-on effect of incarceration on HIV treatment adherence.

*“… during the time people were arrested and staying a week in a police cell were difficult times. They do not know that the person is on treatment … A person was staying three days to a week and even a month in a cell. And the police cannot organize for a person to go to the hospital to receive drugs, that is not possible. And because of people we are shy, we cannot disclose that we are on medication … So, it was difficult during this COVID-19 as others were locked up for a month and they were labelled as defaulters when they were not”.* Female, 30–40 years, Telegram Text.

Ironically, as recounted by another participant, overcrowding in police cells further exacerbated the spread of COVID-19 and was contrary to containment measures **.**

*“Police are violent when they meet people who have not worn a mask and instead of telling them to wear a mask, they just hit people and lock them up in cells and demand for them to pay 10,000 to be released…. And people in those cells are at a risk of getting COVID because there are so many of them in one cell”.* Female, 40–50 years , Telegram Text.

***Stigma.*** Experiences of stigma and discrimination during the pandemic varied and some participants reported that the focus on containing the pandemic and the associated fear of transmission, resulted in less stigma towards PLHIV. 

*“During this COVID-19 period, there is not a lot of stigma on people living with HIV because people are more concerned with COVID and those with COVID are the ones stigmatized and discriminated… Stigma has not increased because people are afraid of COVID-19 disease”*. Male, 50–60 years, Telegram Text.

However, this was not the case for all due to the perception that PLHIV were more vulnerable to contracting COVID-19 due to a “weak immune” system and in some instances more predisposed to spreading the virus to others. 

*“During this time of COVID, stigma and discrimination amongst us living with HIV and AIDS is much and people say we are the ones that are spreading the diseases because our immunity is weak, and they say that we are the ones who can easily get the disease and die”*. Female, 30–40 years, Telegram Text.

***Resilience and resourcefulness.*** In the absence of mental health care professionals’, participants were resilient and resourceful in seeking alternative ways to deal with mental health issues such as seeking support from religious activities and leaving “everything in the hands of God”.

*“In terms of psychologists, there are not here in Malawi. I have not heard that a person was assisted by professionals when they were stressed. A lot of people go to prophets and pastors to be assisted but they still have their stresses and worries with them”.* Female, 30–35 years, Telegram Text.

The participant further explained how in the absence of mental health services, she sought alternative methods such as listening to gospel music and regular exercise to alleviate mental health concerns.

*“In terms of how to take care of my mental health… I just listen to music of encouragement, gospel music and l get encouraged. And if am not very worried then l also listen to songs that l dance to, Malawian music. In terms of exercises l like running and then my body is strong and healthy and then l like stretching myself*.” Female, 30–35 years, Telegram Text.

**Figure 3.**
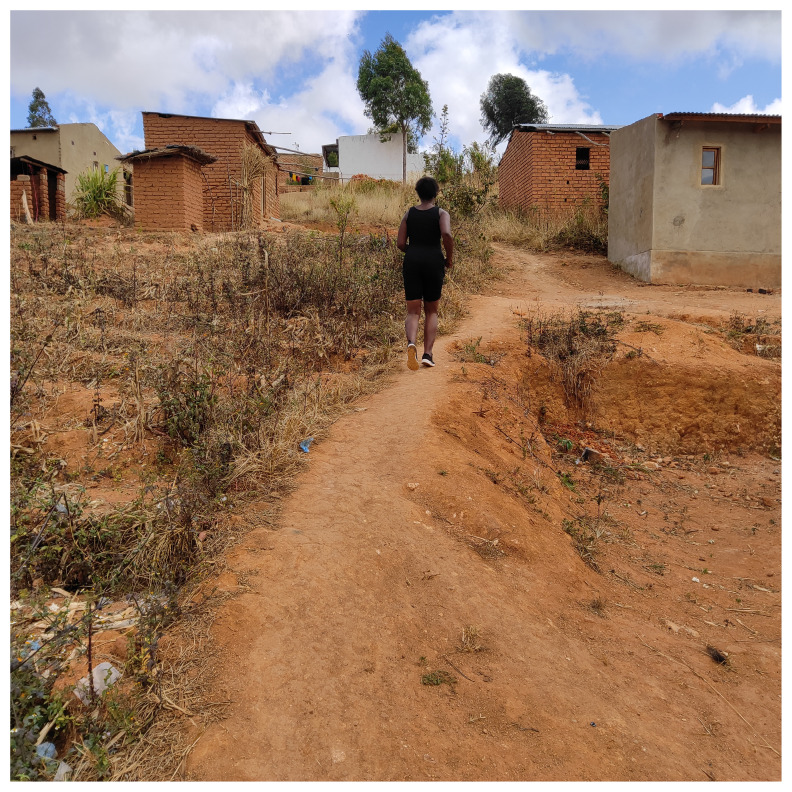
Sometimes I run as one way to make my body strong.

The below image captures exercise to alleviate stress and was captioned “Sometimes I run as one way to make my body strong”. Figure 3

### COVID-19 vaccine

This topic was introduced as the landscape of COVID-19 prevention and treatment evolved including the introduction of the COVID-19 vaccine in Malawi in March 2021. In addition, participants' knowledge of the COVID-19 vaccine, how it works, and the myths and misinformation about the vaccine were explored.

***Knowledge and COVID-19 vaccine uptake.*** All participants had heard about the COVID-19 vaccine and obtained information about the vaccine at health care facilities and via social media, radio and TV.

In addition, most participants reported they were aware of the importance of having the vaccine, especially for PLHIV including those on ARTs. Many also stressed the importance of continuing with preventive measures.

A participant provided a detailed description of the vaccine and what to anticipate in terms of possible side effects:

“ *On the site of injection, we were told that we might experience a little fever we should not be alarmed as the body has been introduced with something new in the body. And we were reminded to still observe the preventative measures and that we should not stop observing them like wearing of the mask, social distance and washing hands. So, this is how the vaccine works, mainly it is to boost the immunity”*. Male, 50–60 years, Voice Note Transcript.

***Vaccine hesitancy.*** Despite misinformation and conspiracy theories circulating in the community, all participants apart from two, had been vaccinated.

However, an unvaccinated participant further reflected on how he had obtained more information from healthcare providers to dispel myths about the vaccine and was considering getting vaccinated: 

*“My perception is that, now that we have been educated by health care workers, we know that all these myths that were said by people were false. And when they start to get people vaccinated again l will get vaccinated*.” Male, 20–30 years, Telegram Text.

***Community views and beliefs around the COVID-19 vaccine.*** Myths, misinformation, mistrust and conspiracy theories around the vaccine and its properties ranged from causing infertility, being a form of population control, a “mark of the beast and Satanism,” to surveillance through 5G monitoring. It is interesting to note that most of the conspiracy theories were similar to conspiracy theories circulating globally during the pandemic.

Religious-themed vaccine misinformation abounded and almost all participants referred to beliefs about the vaccine containing the “mark of the beast” and “being sealed with the number 666”. According to participants, traditional and religious leaders supported the belief that the vaccine was linked to Satanism.

“ *Traditional healers like to mislead people by spreading lies that the vaccine is satanic. If you take the vaccine, then you will not be able to have children. Traditional healers are the only ones that have medicine to protect you from COVID-19. Others say that the vaccine is useless, and it does not work; it is in existence for white people to do business.”* Male, 50 – 60 years, Telegram Text.

 A participant further explained religious references disseminated by religious leaders discouraging people to get vaccinated:

*“Most churches are not encouraging their members to get the vaccine because they are linking it to demonic realms that it is satanic. They are saying that once one gets the vaccine it means that you have been sealed with the number 666 and that a lot of people have been discouraged to get the vaccine*.” Female, 30–35 years, Telegram Text.

The impact of rumors and conspiracy theories on vaccine hesitancy was noted by many individuals.

“ *According to me and with my experience, l think the vaccine is very important but the risk is people did not get vaccinated because of the rumors about the vaccine and this makes people fearful hence those that have been vaccinated are few*”. Female, 20–30 years, Telegram Text. 

***Population control.*** Rumors around government sponsored population control emerged, impacting negatively on vaccine uptake.

“ *It was very difficult with a lot of misconceptions like the government wants to use the vaccine to kill some of its citizens, to reduce the population, or maybe it will make them disabled, or some were even saying that it might be some sort of family planning. There were a lot of talk around the vaccine and its uptake was affected. People were afraid to get vaccinated.”* Male, 50–60 years, Voice Note Transcript.

Linked to population control was the notion of monitoring people through the insertion of chips linked to 5G networks to track people’s movements.

*“The vaccine is a means of "inserting in our bodies" the "5G Micro-chip" - whatever it is, the peddlers of the news have the explanation”.* Male, 60–65 years, Telegram Text.

***Access and vaccination.*** Most participants had not experienced refusal of services due to not being vaccinated. However, one participant reported that friends were refused health care services as it was considered mandatory for PLHIV to be vaccinated. This was reported by one participant only and did not appear to be a regular practice.

“ *Not necessarily me, but my fellow friends on ARV's at Hospital X in Kasungu are being refused any health care service because the management has made it mandatory for any person living with HIV.”* Male, 40–50 years, Telegram Text. 

In contrast, others reported that healthcare access was not contingent on vaccine uptake and people were given a choice whether to be vaccinated.

“ *At the hospital they are not refusing anyone from accessing services because they are not vaccinated against COVID-19. They are providing services to anyone whether vaccinated or not vaccinated, they only give advice for those not vaccinated to get vaccinated and it’s up to the person to vaccinate or not.*” Male, 40–50 years, Telegram Text. 

## Discussion

Whilst community-based participatory research has recently been used amongst PLHIV and informal caregivers in Malawi ^19^, the strength of this study is that we were able to continue to engage with study participants during the different waves of the pandemic and adapt to changes as the pandemic progressed and then subsided.

Similar to findings from other studies ^12,27^, participants in this study reported experiencing disruptions in HIV treatment and prevention services at the onset of the pandemic. However, as observed elsewhere, rapid adjustments such as multi-month ART dispensing and reduced clinical visits were implemented to mitigate the impact of COVID-19 restrictions. These adaptations were crucial in ensuring the continuity of ART, HIV testing, and other preventive services, leading to a steady recovery in many countries by the end of 2020 ^7, 12, 21^. Nonetheless, not all PLHIV reported positive experiences with these changes in service provision, echoing findings from other contexts ^28, 29^. Negative experiences, as seen in Malawi, were linked to limited interaction with healthcare providers and inadequate follow-up on medication side effects.

Interestingly, participants in this study were reluctant to access health facilities due to the fear of COVID-19 testing or vaccination, while in other studies, the main concerns of PLHIV were the risk of infection or the potential disclosure of their HIV status ^29, 30^. State sponsored violence and the reported refusal of health care services without a face mask were more than likely added reasons for avoiding health care facilities. 

Beyond access and utilization of HIV services, COVID-19 restrictions exacerbated structural inequalities experienced by PLHIV, impacting their treatment adherence and well-being ^14^. In line with findings from Malawi, food insecurity was reported globally among PLHIV, attributed not only to movement restrictions but also to the financial implications of COVID-19 preventive measures on income and employment ^11, 14, 29^. Additionally, participants in Malawi highlighted the impact of limited access to clean drinking water on their ability to adhere to ART due to disruptions in borehole maintenance. While not widely reported in other COVID-19 studies, the importance of basic infrastructure such as water and sanitation for PLHIV has been established, not only for ART adherence but also for the prevention of opportunistic infections ^31, 32^. This finding underscores the heightened risk of poor living conditions faced by PLHIV ^14^, particularly in low-income countries like Malawi ^29^. 

Consistent with findings in sub- Saharan Africa including Malawi, this study revealed varied understanding of "U=U", suggesting knowledge gaps regarding the implications of HIV treatment on reducing viral load and transmission which could contribute to stigma experienced by PLHIV, fueled by community perceptions not supported by evidence ^33^. Whilst there were knowledge gaps pertaining to HIV treatment literacy, participants throughout the data collection process undertook their own “education” highlighting the co- learning process where participants had the opportunity to independently participate in the learning and research process.

PLHIV have an increased likelihood of experiencing poor mental health, including stress, depression and anxiety ^34^. Social isolation, loneliness, lack of support networks, and stigma during the pandemic exacerbated these conditions among PLHIV ^13, 14^. Stigma emerged as a significant issue in this study, with some participants noting a reduction in stigma toward PLHIV as attention shifted toward COVID-19 infections, while others reported experiencing intersecting stigmas. A study in the United States (US) echoed similar findings, with some PLHIV fearing compounded discrimination while others expected a reduction in HIV-related stigma as COVID-19 infections became more widespread ^35^.

Experiences of violence and abuse of power by the police heightened mental health issues amongst study participants. The pandemic period in Malawi was shaped by political instability leading to a national election in June 2020. Mistrust towards the government led to people’s non-compliance with COVID-19 restrictive measures, with several demonstrations being held across the country ^36^ which could have fueled misuse of power. Reported police brutality highlighted the increased vulnerability of PLHIV. This was aggravated by PLHIV fear of stigma if their status was disclosed, even when at great risk of exposure and facing higher chances of developing severe COVID-19 disease.

Despite the lack of mental health care services, participants found alternative ways to manage their mental health and well-being. These coping strategies such as spirituality, prayer and exercise align with those identified in a scoping review on the impact of COVID-19 on the mental health of PLHIV ^37^. Additionally, prior experience living with HIV may have made PLHIV somewhat resilient to the impact of COVID-19, with resilience emerging as a key adaptive coping mechanism to minimize adverse effects on mental health and well-being ^14^. 

Another interesting parallel or lesson learned can be drawn between the HIV and COVID-19 pandemics regarding the role of community structures and actors. Historically, community actors such as community health workers and community-based organizations have played a crucial role in providing HIV services outside the traditional health facility setting ^38^. It is important for government policy makers and public health providers and stakeholders to engage more with civil society and community-based organizations which could have important lessons for future pandemics or public health emergencies.

Evidence from the COVID-19 pandemic has shown that community involvement was vital in the response to the pandemic, particularly in integrating COVID-19 and HIV services. This includes supporting PLHIV in continuing ART, raising awareness about COVID-19 vaccination, and providing HIV preventive products such as condoms and HIV self-test kits ^38^. Aligned with this, community-based participatory research offers promising results with vulnerable populations. A study using a community-based participatory research approach to promote well-being among informal caregivers of PLHIV in Malawi showed high adherence to advisory health messages and positive changes in their roles as caregivers to PLHIV, with the full participation of all members in the approach design ^19^.

A scoping review found good general knowledge about COVID-19 in the sub-Saharan Africa region; however, significant gaps in the attitude and practice towards COVID-19 were found ^39^. Similarly, in our study, vaccine uptake and reported hesitancy followed a similar pattern-participants had knowledge about the vaccine, but not all had been vaccinated.

The relationship between misinformation and COVID-19 vaccine hesitancy is complex and medical and scientific approaches may not be sufficient to prevent misinformation ^23, 40^. A systematic review and meta-analysis on COVID-19 vaccine coverage among adult PLHIV found that reasons for low vaccine uptake among adult PLHIV included COVID-19-related medical mistrust, distrust in common sources of vaccine-related information, and reliance on social media for COVID-19 information ^41^. Reliance on social media and other sources of information was evident in our study and participants used the same religious themed phraseology, “mark of the beast” and “666” when reporting on religious based opposition to the vaccine as reported in the US. Conspiracy theories around tracking of personal freedoms with 5 G technology was similarly reported in the US ^40^. Fear and misinformation are likely attributed to fear and global uncertainty during the pandemic and the speed at which vaccines were developed with little time to adequately prepare global communities.

### Limitations

This study has some limitations. The research was conducted in areas where community- led monitoring was taking place and the findings may not be generalizable to other parts of the country, where access to health services might be different.

Working with different forms of data collection including visual methods (photos) without being able to fully engage with participants during the different stages of the pandemic might have led to a stronger focus on the text messages than the visual images. In the next phase of the study, we plan to dedicate more time to the photo images and their closer interpretation with study participants.

## Conclusions

During the onset of the COVID-19 pandemic, understanding the extent of its impact on PLHIV was difficult. The data captured via “Life Maps” provides nuanced, complex, and specific information about the intersection of the two pandemics and their effects on the lives of PLHIV. This information was valuable not only for adapting health delivery approaches promptly, such as the implementation of multi-month dispensing, but also for creating a historical record that can inform future pandemic preparedness and response plans.

At a time when person-centered research and community engagement are top priorities for HIV organizations, including UNAIDS (whose 2023 World AIDS Day slogan was “Let Communities Lead” ^42^, the “Life Maps” methodology offers key opportunities and has the potential to be applied to a variety of other topics and issues, not just in the health sector, but other fields including education, climate change, and humanitarian responses.

## Ethical approval statement and consent to participate

Ethical approval was obtained from the National Health Sciences Research Committee of Malawi (approval number 2267) 12 March 2021. All study participants were provided with written explanations of the work, and thereafter gave their informed consent via signature. Confidentiality and anonymity were ensured, and all participants were given agreed upon pseudonyms to protect their identity. All data were closely controlled and stored in locked files and password protected computer files. All participants were provided a monthly stipend of 24,500 Malawian Kwacha to compensate for their time. Additional written informed consent for the publication of the participants’ images created for the project was obtained from all participants.

### Ethical and security consideration

All data underlying the results are available as part of the article and no additional source data are required. The audio files and transcripts will not be shared because the files may contain sensitive data and might be identifiable.

### Data protection issue

Data will only be made available by the corresponding author on a per request basis from an established author at an established institution, including the purpose for which the data is sought and will be assessed on a case-by-case basis. All identifiers will be removed to protect confidentiality.

## Consent to publish

Written informed consent for publication of the participants details and their images was obtained from all study participants. All data has been de- identified. 

## Data Availability

All data underlying the results are available as part of the article and no additional source data are required. The audio files will not be shared because the files may contain sensitive data and might be identifiable. Data will only be made available by the corresponding author on a per-request basis from an established author, including the purpose for which the data is sought, on a case-by-case basis. All identifiers will be removed to protect confidentiality. Supplementary material has been uploaded in the ZivaHub Open UCT with the DOI link https://doi.org/10.25375/uct.26233337.v1 This article complies with the reporting guidelines for qualitative research using the Standards for Reporting Qualitative Research (SRQR). https://doi.org/10.25375/uct.26233337.v1
