# Supplementary material for: "Life mapping" exploring the lived experience of COVID-19 on access to HIV treatment and care in Malawi
Source: Gates Open Res. 2024 Sep 24;8:70. Originally published 2024 Jul 19. [Version 2] doi: 10.12688/gatesopenres.15927.2 (PMC11781794; doi:10.12688/gatesopenres.15927.2)
Supplement: Peer review history

# Reviewer report: Reviewer response for version 2

**DOI:** 10.21956/gatesopenres.17686.r38824

**Published:** 2024-12-24

**Authors:** Anggraini Alam

**Affiliations:** 1 Universitas Padjadjaran-Hasan Sadikin General Hospital, Bandung, Indonesia

This article has explained well the background and objectives of the study which are in line with the results, discussion and conclusion sections. >95% of the citations used are the latest literature 2020-2024, some come from older literature but are still related to support.

 In accordance with the title, to find out the life experience of HIV patients during the COVID-19 pandemic, the selection of a qualitative research design is appropriate and correct in its implementation because it uses a community-based project that has also been previously established.

 The details of the method and analysis have been clearly described by the author, along with limitations and potential for further studies so that they can be an opportunity for other researchers.

 No quantitative analysis was performed so statistical analysis is not applicable to this article.

 The source data found in this article are figures that can be used to correlate with the information that has been provided based on the results of the study obtained.

 The conclusion made by the author has summarized all the data obtained from the study.

 Regarding limitations, the author can explain the efforts that have been made to avoid or minimize their impact on the study conducted. One of them is mentioned that the selected population may not represent other parts of the country, it is better to explain the reasons for choosing a community-led area compared to other areas.

Is the work clearly and accurately presented and does it cite the current literature?

Yes

If applicable, is the statistical analysis and its interpretation appropriate?

Not applicable

Are all the source data underlying the results available to ensure full reproducibility?

Yes

Is the study design appropriate and is the work technically sound?

Yes

Are the conclusions drawn adequately supported by the results?

Yes

Are sufficient details of methods and analysis provided to allow replication by others?

Yes

Reviewer Expertise:

Paediatric, Infectious and Tropical Diseases

I confirm that I have read this submission and believe that I have an appropriate level of expertise to confirm that it is of an acceptable scientific standard.

# Response

**Published:** 2024-12-27

**Authors:** Jane Harries

**Affiliations:** University of Cape Town, Rondebosch, Western Cape, South Africa

Thank you for your positive review. In terms of the comment for limitations, the community led monitoring sites in Malawi were chosen as they formed part of the community led monitoring Citizen Science project as explained in the methods and background section of the manuscript.

# Reviewer report: Reviewer response for version 2

**DOI:** 10.21956/gatesopenres.17686.r38122

**Published:** 2024-10-24

**Authors:** Warren Parker

**Affiliations:** 1 Centre for Communication Media and Society, University of KwaZulu-Natal, Durban, KwaZulu-Natal, South Africa

I have reviewed the changes made by the authors and the article is accepted based on these changes

Is the work clearly and accurately presented and does it cite the current literature?

Yes

If applicable, is the statistical analysis and its interpretation appropriate?

Not applicable

Are all the source data underlying the results available to ensure full reproducibility?

No source data required

Is the study design appropriate and is the work technically sound?

Yes

Are the conclusions drawn adequately supported by the results?

Yes

Are sufficient details of methods and analysis provided to allow replication by others?

Yes

Reviewer Expertise:

NA

I confirm that I have read this submission and believe that I have an appropriate level of expertise to confirm that it is of an acceptable scientific standard.

# Response

**Published:** 2024-10-24

**Authors:** Jane Harries

**Affiliations:** University of Cape Town, Rondebosch, Western Cape, South Africa

Thank you for approving the revised version of the article.

# Reviewer report: Reviewer response for version 1

**DOI:** 10.21956/gatesopenres.17285.r37761

**Published:** 2024-08-29

**Authors:** Warren Parker

**Affiliations:** 1 Centre for Communication Media and Society, University of KwaZulu-Natal, Durban, KwaZulu-Natal, South Africa

This article addresses the intersections between COVID-19 and HIV treatment and care in Malawi through applying a relevant participatory research methodology — Life Mapping.

 The article is overall very well written with a sound and logical sequence in framing and presenting the data. 

 While the background provides an overview HIV and COVID-19 in Malawi, it would be useful to also consider expanding the background information from related literature so as to frame the forthcoming findings. For example, what is known about the intersections between COVID-19 and HIV treatment and care in similar southern and East African settings. Some of this information is included in the discussion, and could be brought forward. This helps to understand the added value of the findings, and whether there are new insights emerging, or that the findings reinforce experiences in other similar settings (which can then be explored further in the discussion section).

 A few concepts in the background should be explained more definitively. For example, regarding participatory research, the phrasing "community members are placed as equal partners in all phases of the research process" is an over-elaboration. Participatory research processes do not remove the inevitable unequal power relations between researchers and community participants. It would be preferable to refer to this process as a "co-learning process", whereby researchers and participants recognize and appreciate the power differentials, and where researchers and community participants have an opportunity to engage with the knowledge that they are producing independently or collectively. This point can be later elaborated in the discussion (for example, where participants "undertook their own education").

 Community Led Monitoring should be defined more clearly when it is referred to.

 The phrasing "...and an innovative research methodology we have called 'life mapping'… infers unique origination of this term in this study. 'Life mapping' has been used by other researchers in various settings and his previous literature should be referred to. Any variations in the methods of the present application of the approach can then be explained. 

 Add "who are the" after (MANERELA+).

 It is suggested that in the abstract and later in the discussion, the phrasing "this is the first study conducted..." be removed. There is no need to rank the study in this way. 

 The discussion is overall sound, and is closely linked to the findings. 

 The conclusions provide summative insights into the relevance of life mapping and person-centered research methods. However, it would also be important to consider the implications of the findings for public health practitioners and government policy makers. Notably, how the two diseases intersected, and how negative outcomes of these intersections could be better addressed in future epidemic and pandemic scenarios.

 For example, multi-month dispensing is considered to be an innovative approach, yet in this study important limitations arise. Also, creating the circumstances for corruption (harsh regulations) led to heavy-handed policing requiring already impoverished people living with HIV to hand over bribes or face incarceration where their treatment was disrupted.

 In contrast, it is heartening to see that, apart from one example, not being COVID-19 vaccinated did not impact access to health care. Additional literature in similar settings could be discussed to reflect whether this was the case elsewhere.

Is the work clearly and accurately presented and does it cite the current literature?

Yes

If applicable, is the statistical analysis and its interpretation appropriate?

Not applicable

Are all the source data underlying the results available to ensure full reproducibility?

No source data required

Is the study design appropriate and is the work technically sound?

Yes

Are the conclusions drawn adequately supported by the results?

Yes

Are sufficient details of methods and analysis provided to allow replication by others?

Yes

Reviewer Expertise:

International experience in HIV and participatory research, and COVID-19 response.

I confirm that I have read this submission and believe that I have an appropriate level of expertise to confirm that it is of an acceptable scientific standard, however I have significant reservations, as outlined above.

# Response

**Published:** 2024-09-19

**Authors:** Jane Harries

**Affiliations:** University of Cape Town, Rondebosch, Western Cape, South Africa

**Response to Reviewer Report**

**29 Aug 2024 | for Version 1**

 Thank you for the positive and insightful comments and for suggestions to strengthen the article titled: “Life Mapping” Exploring the lived experience of COVID-19 on access to HIV treatment and care in Malawi.

- Reviewer: What is known about the intersections between COVID-19 and HIV treatment and care in similar southern and East African settings. Some of this information is included in the discussion and could be brought forward.
- **Response**: Thank you for the suggestion however we wanted to focus on Malawi in the introduction and bring in other settings as a comparison into the discussion and have left in the discussion to highlight where results differed or had resonance in other settings.
- Reviewer: The phrasing "community members are placed as equal partners in all phases of the research process" is an over-elaboration. Participatory research processes do not remove the inevitable unequal power relations between researchers and community participants. 
- **Response:** We recognise the inevitable unequal power relations between researchers and community and have removed “equal partners” and replaced with “ **partners”**.   

We have added co- learning processes in the discussion section and have added.

**“Whilst there were knowledge gaps pertaining to HIV treatment literacy, participants throughout the data collection process undertook their own “education” highlighting the co- learning process where participants had the opportunity to independently participate in the learning and research process”.**

- Reviewer: Community Led Monitoring should be defined more clearly when it is referred **Response:**  We have defined community led monitoring more clearly in the background: **“** **Community led monitoring in this study context, can be described as a community monitoring and research initiative that gathers data on access to and quality of HIV treatment. CLM aims to streamline and standardize HIV treatment access data collected by communities and uses this data to guide advocacy efforts and promote accountability (20)”.**
- Reviewer: The phrasing "...and an innovative research methodology we have called 'life mapping'… infers unique origination of this term in this study. 'Life mapping' has been used by other researchers in various settings and his previous literature should be referred to. Any variations in the methods of the present application.
- **Response: **We did expand further on the term “life mapping” in the methods section and have now clarified that it was an adaptation of the term and referenced Flaherty J, Garratt E. Life history mapping: Exploring journeys into and through housing and homelessness. Qual Res [Internet]. 2022. We have added **“We used and adapted the term “Life Mapping” informed by a life history approach in which participants were able to narrate their everyday life experiences through textual and visual presentations on diverse topics during and in the aftermath of the COVID-19 pandemic** **(23)** **.** **”**
- Reviewer: Add "who are the" after (MANERELA+).
- **Response**: “ **who are the” **has been added to after MANERELA+
- Reviewer: It is suggested that in the abstract and later in the discussion, the phrasing "this is the first study conducted..." be removed. There is no need to rank the study in this way.
** **
-  
**Response**: “ **This is the first study conducted**” has been removed in the abstract and discussion.
- Reviewer: It would also be important to consider the implications of the findings for public health practitioners and government policy makers. Notably, how the two diseases intersected, and how negative outcomes of these intersections could be better addressed in future epidemic and pandemic scenarios.
- **Response**: In the discussion we do highlight the important role that community-based organizations can play in response to pandemics and integrating HIV services but were not that explicit of the role that government policy makers and other public health stakeholders can play. We have now added: **“It is important for government policy makers and public health providers and stakeholders to engage more with civil society and community-based organizations which could have important lessons for future pandemics or public health emergencies”**.
